# Correlation of hemoglobin with osteoporosis in elderly Chinese population: A cross-sectional study

**DOI:** 10.3389/fpubh.2023.1073968

**Published:** 2023-04-13

**Authors:** Yichen Liu, Yue Zeng, Jun Lu, Xiaoya Zhang, Zikai Zhang, Huijuan Li, Peipei Liu, Bin Ma, Yiqun Gu, Lige Song

**Affiliations:** ^1^Department of Endocrinology, Shanghai Tongji Hospital, School of Medicine, Tongji University, Shanghai, China; ^2^Institute of Osteoporosis and Metabolic Bone Diseases, School of Medicine, Tongji University, Shanghai, China; ^3^Department of Endocrinology and Metabolism, Putuo Hospital, Shanghai University of Traditional Chinese Medicine, Shanghai, China; ^4^Division of Science and Research, Shanghai Tongji Hospital, School of Medicine, Tongji University, Shanghai, China; ^5^Division of Spine, Department of Orthopedics, Shanghai Tongji Hospital, School of Medicine, Tongji University, Shanghai, China; ^6^Ganquan Community Health Service Center, Shanghai, China

**Keywords:** hemoglobin, osteoporosis, bone mineral density, elderly population, Chinese

## Abstract

**Introduction:**

In the elder population, both low hemoglobin (Hb)/anemia and osteoporosis (OP) are highly prevalent. However, the relationship between Hb and OP is still poorly understood. This study was to evaluate the correlation between Hb and OP in Chinese elderly population.

**Methods:**

One thousand and sisty-eight individuals aged 55–85 years were enrolled into this cross-sectional study during June 2019–November 2019. Data on the demographics and clinical characteristics were recorded. Detections of complete blood count, liver/kidney function, glucose metabolism and lipid profile, and thoracolumbar X-ray were performed, and bone mineral density (BMD) at lumbar spine 1–4, femur neck, and total hip was measured by dual-energy X-ray absorptiometry (DXA). Univariate and multivariate linear regression analyses were employed to evaluate the correlation between Hb with BMD T-score. Logistic regression analysis was performed to access the correlation between different Hb levels and the odds ratio (OR) for OP.

**Results:**

Compared with non-OP group, OP patients had lower level of Hb. Univariate linear regression analysis indicated Hb level was positively related to the BMD of lumbar spine 1–4, femur neck and total hip, and this relationship remained after adjusting confounding variables [gender, age, body mass index (BMI), diabetes mellitus (DM) and morphological vertebral fracture]. Logistic regression analysis showed the ORs for OP decreased with the increase of Hb. Compared with the subjects with the lowest quartile of Hb, the OR for OP in the highest quartile group was 0.60 (0.41–0.89) after adjusting for gender, age and BMI, and the OR for OP was 0.62 (0.41–0.92) after further adjustment for gender, age, BMI, DM, and lipid indexes.

**Discussion:**

In conclusion, Lower Hb level is related to lower BMD in the elderly population. However, whether Hb level could be used to predict the risk of OP needs to be further determined in more longitudinal clinical studies.

## 1. Introduction

Along with the aging of society, osteoporosis (OP) and anemia become more and more popular, which may be related to the prevalence of malnutrition in the aged population (1, 2). OP is a metabolic skeletal disease characterized by the decreased bone mineral density (BMD) and microarchitectural deterioration with increased bone fragility and susceptibility to fractures, which may increase the disability and mortality (3, 4). OP, the most common metabolic bone disorder, brings major burden to the public health and affects an enormous number of people (5). According to the statistics from the International Osteoporosis Foundation (IOF), one in three women and one in five men older than 50 years will experience osteoporotic fracture in their lifetime (6). There are many causes for the decreased BMD in the elder population, including reduction of sex steroid (7), lower insulin-like growth factor-1(IGF-1) (8), inadequate physical activity (9), and improper nutrition status (10). In addition, nutritional deficiency due to reduced dietary intake and/or inadequate absorption of particular nutrients has been identified as an important cause of osteopenia and OP (11).

Hemoglobins (Hbs) are large and complex protein molecules, and the Hb level is related to nutrients from dietary intakes (12). Animal experiments have shown that lower Hb level is related to the impaired bone turnover and poor bone strength (13–15). Dietary iron deficiency in Wistar rats can induce the decrease of Hb concentration, accompanied by decreased serum osteocalcin concentration, bone mineral content, BMD, and mechanical strength of the femur (14). Thalassemia and sickle cell anemia are two hematopoietic disorders. The trabecular bone volume, trabecular number, trabecular thickness, cortical thickness, and cortical area reduce significantly in both heterozygous and homozygous thalassemia mice (th3) (16). These bone abnormalities in thalassemia may be induced by ineffective erythropoiesis, iron overload, chelation, and endocrine dysfunction secondary to iron overload (17, 18). In addition, abnormal bone microarchitectural and biomechanical properties have also been identified in sickle cell anemia mice (19). Comparing to wild-type mice, transgenic sickle cell anemia mice displayed significantly fewer and deteriorated geometry trabeculae, decreased cortical thickness, and lower elastic modulus of the cortical bone (19). These indicate that lower Hb level and anemia are related to the abnormal bone phenotypes in animals.

There is evidence showing that lower Hb level is related to the lower BMD. An Italy population-based study on 950 elderly individuals showed that the individuals with lower Hb levels or anemia had lower bone density on the peripheral quantitative computed tomography (pQCT), especially in the cortical bone (20). Similar results were reported in another Italy study on the elderly population (>75 years) when BMD was detected by ultrasound bone densitometer (21). A Korean cross-sectional study involving 13,127 subjects (>20 years) also showed that Hb level was positively related to the lumbar spine and femoral neck BMD on dual-energy X-ray absorptiometry (DXA) in men (22). Concerning the possible biological mechanisms between Hb and BMD, the bone-derived hormone fibroblast growth factor 23 (FGF-23), mainly produced by osteoblasts and osteocytes, can regulate erythropoiesis and may be an important link between bone metabolism and erythropoiesis (23). Besides, another study proposed that extracellular acidification and oxidative stress under hypoxemia caused by anemia could increase bone resorption and lead to lower BMD (24). Nevertheless, a prospective longitudinal study from United States failed to show the association between Hb level and lumbar spine or total hip BMD in the community-dwelling older individuals (25). Besides BMD, some studies have also focused on the relationship between serum Hb level and fracture risk. A prospective cohort study showed that the increased fracture risk associated with anemia ranged from 7 to 38% across the fracture sites in the United States. postmenopausal women of diverse racial and ethnic backgrounds (26), but some other studies showed the U-shaped relationship between Hb level and hip fracture risk (27).

There were few studies investigating the relationship between Hb and OP in China. A cross-sectional study which included 495 type 2 diabetes mellitus (T2DM) patients showed that Hb levels were associated with the presence of OP in male patients, especially in those with aged 50 years and older (28). Another retrospective study conducted in China demonstrated that the Hb level in the OP group were higher than those without OP, and moreover, BMD was negatively correlated with Hb level in postmenopausal women (29). To further confirm the relationship between Hb level and OP in aged Chinese individuals, we conducted a cross-sectional study with larger samples than the published study.

## 2. Materials and methods

### 2.1. Study population

This was a cross-sectional study conducted from June 2019 to November 2019. The participants in this study were recruited from individuals who underwent routine physical examination at Ganquan community and Yichuan community in Putuo District, Shanghai, China. Questionnaire including name, gender, age, birth of data, race, home address, weight, and height was collected during visit. The inclusion criteria were as follows: (1) 55–85 years old; (2) BMI between18.5 and 35.0 kg/m^2^; (3) normal liver and renal function; and (4) never use of drugs that can affect bone metabolism such as anti-osteoporotic drugs, glucocorticoid, thyroid hormones, estrogen, and thiazolidinedione. The exclusion criteria were as follows: (1) a history of gastrointestinal surgery; (2) a history of Hb disease (e.g., sickle cell anemia, thalassemia); (3) a history of diseases that can affect Hb levels (e.g., diabetes insipidus and parathyroid storm); (4) a history of major cardiovascular and cerebrovascular diseases; (5) autoimmune diseases; (6) malignant tumors; (7) mental diseases; (8) Non-menopausal women; and (9) with unhealthy lifestyle habits (e.g., addiction to smoking and excessive drinking). A total of 1,068 participants (449 males and 619 females) were recruited into this study. OP was diagnosed according to the AACE/ACE primary osteoporosis guideline (2020) (2). Written informed consent was obtained from each participant. This study was approved by the Ethics Committee of Shanghai Tongji Hospital.

### 2.2. Demographics and clinical characteristics

Data on demographics including age, height, weight, body mass index (BMI), and clinical characteristics (diseases, medications, etc.) were recorded. Physical examinations were performed by trained medical staffs following standardized procedures.

### 2.3. Complete blood count and biochemical parameters

Fasting blood samples were obtained and complete blood count [red blood cell (RBC) count, Hb, mean corpuscular volume (MCV), mean corpuscular hemoglobin (MCH), mean corpuscular hemoglobin concentration (MCHC), and white blood cell (WBC) count] was measured using an automatic blood analyzer (SYSMEX XN3000) immediately. Serum and plasma were stored at −80°C for further automatic biochemical assays, including glucose metabolic indices, lipid profile [triglyceride (TG), total cholesterol (TC), low-density lipoprotein-cholesterol (LDL-C), and high-density lipoprotein-cholesterol (HDL-C)], and liver and kidney function. All laboratory examinations were performed in Shanghai Tongji Hospital, Shanghai, China. Considering gender difference, patients were divided into four groups according to Hb quartiles as follows: Quartile 1, ≤143.0; Quartile 2, 143.1–151.0; Quartile 3, 151.1–159.6; and Quartile 4, >159.6 g/L for men; and Quartile 1, ≤130.0; Quartile 2, 130.1–137.0; Quartile 3, 137.1–143.0; and Quartile 4, >143.0 g/L for women, separately.

### 2.4. Thoracolumbar spine X-ray

The radiographs of the spine (standing long-cassette coronal and lateral views) of each individual were evaluated with the Genant visual semi-quantitative method independently by three radiologists. The lateral view should include at least T4 to L5.

### 2.5. BMD by dual-energy X-ray absorptiometry

Bone mineral density at lumbar spine 1–4, femur neck and total hip were detected by dual-energy X-ray absorptiometry (DXA, HOLOGIC Discovery; coefficient of variation <1%).

### 2.6. Statistical analysis

Kolmogorov–Smirnov test was used to assess the distribution of continuous variables. Data were expressed as median (interquartile range, IQR) or mean ± SD for continuous variables or as frequency (%) for categorical variables. The differences between continuous variables were examined using the Mann–Whitney U test or Student’s *t*-test; categorical variables were tested using the Chi-square test.

Multivariate linear regression model was employed to analyze the association of BMD T-score at each site with clinical characteristics and results from blood examinations. Logistic regression model was used to estimate the odds ratios for OP according to different Hb levels/categories. All analyses were adjusted for gender, age, and BMI (Model 1), and then for gender, age, BMI, and diabetes mellitus (DM; Model 2), and further for gender, age, BMI, DM, TG, TC, LDL-C, and HDL-C. A value of two-sided *p* < 0.05 was considered statistically significant.

## 3. Results

### 3.1. Clinical characteristics

The enrolled individuals (*n* = 1,068) were divided into osteoporotic (OP) and non-osteoporotic (non-OP) groups according to the guideline from the IOF (30). Subjects in the OP group had significantly older age (68.81 ± 4.96 vs. 67.81 ± 5.57 years, *p* = 0.004) and lower BMI (23.15 ± 3.88 vs. 24.40 ± 3.47 kg/m^2^, *p* < 0.001) when compared with those in the non-OP group. Furthermore, the prevalence of DM was lower (20.54 vs. 30.05%, *p* = 0.001) and the rate of morphological vertebral fracture was higher (78.85 vs. 45.22%, *p* < 0.001) in the OP group as compared to the non-OP group.

Red blood cell count, Hb, and mean corpuscular hemoglobin (MCH) were markedly lower in the OP group than in the non-OP group (4.56 ± 0.51 × 10^12^/L vs. 4.78 ± 0.50 × 10^12^/L, *p* < 0.001; 137.68 ± 12.35 vs. 145.40 ± 13.15 g/L, *p* < 0.001 and 30.37 ± 1.83. vs 30.65 ± 1.72 pg., *p* = 0.015). Moreover, as compared to the subjects in the non-OP group, subjects in the OP group had lower fasting plasma glucose (FBG; 5.51 ± 1.28 vs. 5.80 ± 1.45 mmol/L, *p* = 0.001) and higher TC (5.41 ± 1.10 vs. 5.05 ± 1.07 mmol/L, *p* < 0.001), LDL-C (3.11 ± 0.94 vs. 2.90 ± 0.95 mmol/L, *p* = 0.001) and HDL-C (1.51 ± 0.44 vs. 1.33 ± 0.40 mmol/L, *p* < 0.001) but lower TG (1.64 ± 0.93 vs. 1.79 ± 1.20 mmol/L, *p* = 0.020; Table 1).

**Table 1:** Demographic and clinical characteristics of individual with/without osteoporosis.

Variable	Non-OP group (*n* = 659)	OP Group (*n* = 409)	*p* value
Gender (men/women)	361/298	88/321	<0.001^***^
Age (years)	67.81 ± 5.57	68.81 ± 4.96	0.004^**^
BMI (kg/m^2^)	24.40 ± 3.47	23.15 ± 3.88	<0.001^***^
Diabetes mellitus, *n* (%)	198 (30.05)	84 (20.54)	0.001^**^
Vertebral fracture, *n* (%)	298 (45.22)	321 (78.85)	<0.001^***^
Complete blood count			
RBC count (×10^12^/L)	4.78 ± 0.50	4.56 ± 0.51	<0.001^***^
Hb (g/L)	145.40 ± 13.15	137.68 ± 12.35	<0.001^***^
MCV (fl)	91.56 ± 6.50	91.15 ± 6.66	0.329
MCH (pg)	30.65 ± 1.72	30.37 ± 1.83	0.015^*^
MCHC (g/L)	338.00 (321.00–351.00)	336.00 (321.00–348.00)	0.126
WBC count (×10^9^/L)	5.75 ± 1.30	5.61 ± 1.41	0.102
Lipid profile			
TG (mmol/L)	1.54 (1.00–2.08)	1.44 (1.00–2.00)	0.034^*^
TC (mmol/L)	5.05 ± 1.07	5.41 ± 1.10	<0.001^***^
LDL-C (mmol/L)	2.90 ± 0.95	3.11 ± 0.94	0.001^**^
HDL-C (mmol/L)	1.23 (1.00–1.55)	1.46 (1.09–1.97)	<0.001^***^
Liver and kidney function			
AST (U/L)	20.00 (17.00–24.00)	19.00 (17.00–23.00)	0.208
ALT (U/L)	17.00 (14.00–24.00)	16.00 (12.00–21.00)	<0.001^***^
Serum creatinine (μmol/L)	75.00 (65.00–85.00)	67.00 (59.00–76.00)	<0.001^***^
Bone mineral density (T-score)			
Lumbar spine (L1–L4)	−0.77 ± 1.25	−2.89 ± 0.90	<0.001^***^
Femur neck	−1.44 ± 0.69	−2.65 ± 0.60	<0.001^***^
Total hip	−0.62 ± 0.79	−1.87 ± 0.71	<0.001^***^

Data are expressed as mean ± SD, median (p25–p75), or number (%).

Non-OP group, participants without OP; OP group, participants with OP; BMI, body mass index; RBC, red blood cell; Hb, hemoglobin; MCV, mean corpuscular volume; MCH, mean corpuscular Hb; MCHC, mean corpuscular Hb concentration; WBC, white blood cell; TG, Triglyceride; TC, Total cholesterol; LDL-C, low-density lipoprotein-cholesterol; HDL-C, high-density lipoprotein-cholesterol; AST, aspartate aminotransferase; and ALT, alanine aminotransferase. ^*^p < 0.05; ^**^p < 0.01; and ^***^p < 0.001.

### 3.2. Correlation between Hb and BMD T-score on multivariate linear regression analysis

The correlations of clinical parameters (gender, age, BMI, history of DM, and vertebral fracture) and Hb with BMD T-score were analyzed by multivariate linear regression. As compared to males, the gender of female was negatively related to the BMD T-score at lumbar spine 1–4 (β = −1.156, *p* < 0.001), femur neck (β = −0.494, *p* < 0.001), and total hip (β = −0.513, *p* < 0.001). Age was negatively related to the BMD T-score at the femur neck (β = −0.031, *p* < 0.001) and total hip (β = −0.015, *p* = 0.004). Moreover, BMI and history of DM were positively related to the BMD T-score at lumbar spine 1–4 (β = 0.083, *p* < 0.001 and β = 0.409, *p* < 0.001), femur neck (β = 0.036, *p* < 0.001 and β = 0.244, *p* < 0.001), and total hip (β = 0.054, *p* < 0.001 and β = 0.303, *p* < 0.001), and fracture history was negatively related to the BMD T-score at femur neck (β = −0.178, *p* = 0.004) and total hip (β = −0.267, *p* < 0.001). Most interestingly, Hb level was positively related to the BMD T-score at lumbar spine 1–4 (β = 0.008, *p* = 0.035), femur neck (β = 0.006, *p* = 0.005), and total hip (β = 0.007, *p* = 0.007; Table 2).

**Table 2:** Multivariate linear regression analysis of clinical parameters and BMD T-score.

	BMD of lumbar spine (L1-L4; T-score)		BMD of femur neck (T-score)		BMD of total hip (T-score)	
Covariates	β (95% CI)	*p* value	β (95% CI)	*p* value	β (95% CI)	*p* value
Gender						
Male	Reference					
Female	−1.156 (−1.354, −0.959)	<0.001^***^	−0.494 (−0.611, −0.378)	<0.001^***^	−0.513 (−0.643, −0.384)	<0.001^***^
Age	0.001 (−0.015, 0.016)	0.911	−0.031 (−0.040, −0.022)	<0.001^***^	−0.015 (−0.025, −0.005)	0.004^**^
BMI	0.083 (0.061, 0.105)	<0.001^***^	0.036 (0.023, 0.049)	<0.001^***^	0.054 (0.039, 0.068)	<0.001^***^
DM						
Non-DM	Reference					
DM	0.409 (0.222, 0.595)	<0.001^***^	0.244 (0.134, 0.354)	<0.001^***^	0.303 (0.181, 0.425)	<0.001^***^
Morphological vertebral fracture						
No fracture	Reference					
Fracture	−0.198 (−0.403, 0.006)	0.057	−0.178 (−0.298, −0.057)	0.004^**^	−0.267 (−0.402, −0.133)	<0.001^***^
Hb	0.008 (0.001, 0.015)	0.035^*^	0.006 (0.002, 0.010)	0.005^**^	0.007 (0.002, 0.011)	0.007^**^

BMD, bone mineral density; BMI, body mass index; DM, diabetes mellitus; Hb, hemoglobin; CI, confidence interval. ^*^p < 0.05; ^**^p < 0.01; and ^***^p < 0.001.

### 3.3. Correlation between complete blood count and BMD T-score

Then, the correlation of complete blood count with BMD T-score was further evaluated after adjusting gender, age, BMI, history of DM, and blood lipid profile (TG, TC, LDL-C, and HDL-C). In the Model 1, after adjusting gender, age and BMI, RBC count, and Hb level were positively related to the BMD T-score at lumbar spine 1–4 (β = 0.204, *p* = 0.020 and β = 0.008, *p* = 0.037), femur neck (β = 0.133, *p* = 0.011 and β = 0.006, *p* = 0.005), and total hip (β = 0.132, *p* = 0.023 and β = 0.007, *p* = 0.006). Meanwhile, MCV was negatively related to the BMD T-score at total hip (β = −0.010, *p* = 0.018) and MCHC was positively related to the BMD T-score at total hip (β = 0.007, *p* < 0.001). After further adjusting for DM (Model 2) and blood lipid profile (Model 3), the positively correlation between Hb and BMD T-score remained (Table 3).

**Table 3:** Correlation between blood indices and BMD T-score at lumbar spine1-4, femur neck, and total hip.

	BMD of lumbar spine (L1–L4; T-score)		BMD of femur neck (T-score)		BMD of total hip (T-score)	
Covariates	β	*p* value	β	*p* value	β	*p* value
Model 1: Adjusting gender, age, and BMI						
RBC count	0.204	0.020^*^	0.133	0.011^*^	0.132	0.023^*^
Hb	0.008	0.037^*^	0.006	0.005^**^	0.007	0.006^**^
MCV	−0.008	0.224	0.000	0.957	−0.010	0.018^*^
MCH	−0.002	0.935	0.008	0.568	0.024	0.141
MCHC	0.003	0.116	0.001	0.455	0.007	<0.001^***^
WBC count	−0.001	0.985	−0.006	0.736	0.011	0.583
Model 2: Adjusting gender, age, BMI, and DM						
RBC count	0.190	0.130	0.124	0.017^**^	0.121	0.036^*^
Hb	0.008	0.027^*^	0.006	0.003^**^	0.007	0.004^**^
MCV	−0.005	0.405	0.002	0.651	−0.008	0.049^*^
MCH	0.009	0.715	0.015	0.309	0.032	0.048^*^
MCHC	0.003	0.128	0.001	0.492	0.006	<0.001^***^
WBC count	−0.018	0.574	−0.017	0.373	−0.001	0.957
Model 3: Adjusting gender, age, BMI, DM, TG, TC, LDL-C, and HDL-C						
RBC count	0.170	0.058	0.112	0.035^*^	0.083	0.158
Hb	0.007	0.049^*^	0.006	0.008^**^	0.005	0.033^*^
MCV	−0.007	0.267	0.002	0.550	−0.006	0.153
MCH	0.010	0.679	0.016	0.273	0.035	0.029^*^
MCHC	0.004	0.050	0.001	0.526	0.006	<0.001^***^
WBC count	−0.038	0.244	−0.024	0.203	−0.014	0.519

BMD, bone mineral density; RBC, red blood cell; Hb, hemoglobin; MCV, mean corpuscular volume; MCH, mean corpuscular Hb; MCHC, mean corpuscular Hb concentration; and WBC, white blood cell. ^*^p < 0.05; ^**^p < 0.01; and ^***^p < 0.001.

### 3.4. Relationship of hemoglobin level with odds ratios of OP

In the overall enrolled individuals including men and women, the prevalence of OP in the groups of quartiles 3 and 4 of Hb level was lower than in the group of quartile 1 after adjusting gender, age, BMI, DM, TG, TC, LDL-C, and HDL-C (Table 4), and this was also observed in the groups of quartile 3 and 4 in women and in the group of quartile 3 in men (Table 4). For one SD increase of Hb level, the adjusted OR for OP was 0.73 (0.57–0.92; *p* = 0.008) in women and 0.74 (0.57–0.96; *p* = 0.025) in men (Table 4).

**Table 4:** Relationship between Hb quartiles and risk of OP after gender stratification and adjustment.

	No. of participants	No. of cases	Model 1		Model 2		Model 3	
			Odds ratio (95% CI)	*p* value	Odds ratio (95% CI)	*p* value	Odds ratio (95% CI)	*p* value
Overall								
Quartile1	256	124	1 (reference)		1 (reference)		1 (reference)	
Quartile 2	281	122	0.84 (0.58–1.22)	0.361	0.84 (0.58–1.21)	0.339	0.83 (0.57–1.20)	0.310
Quartile 3	263	83	0.55 (0.37–0.81)	0.002^**^	0.54 (0.37–0.78)	0.002^**^	0.54 (0.36–0.80)	0.002^**^
Quartile 4	248	78	0.60 (0.41–0.89)	0.012^*^	0.60 (0.41–0.90)	0.012^*^	0.62 (0.41–0.92)	0.019^*^
*p* for trend				0.001^**^		0.001^**^		0.001^**^
One SD increase			0.75 (0.63–0.88)	0.001^**^	0.74 (0.63–0.88)	<0.001^***^	0.75 (0.63–0.89)	0.001^**^
Men								
Quartile 1	120	30	1 (reference)		1 (reference)		1 (reference)	
Quartile 2	108	22	0.88 (0.46–1.67)	0.695	0.89 (0.46–1.70)	0.716	0.84 (0.44–1.62)	0.603
Quartile 3	110	13	0.45 (0.21–0.94)	0.034^*^	0.44 (0.21–0.94)	0.034^*^	0.42 (0.19–0.89)	0.024^*^
Quartile 4	111	21	0.87 (0.45–1.69)	0.688	0.88 (0.45–1.71)	0.699	0.79 (0.39–1.57)	0.493
*p* for trend				0.340		0.347		0.225
One SD increase			0.79 (0.62–1.01)	0.057	0.79 (0.61–1.01)	0.059	0.74 (0.57–0.96)	0.025^*^
Women								
Quartile 1	156	94	1 (reference)		1 (reference)		1 (reference)	
Quartile 2	173	100	0.82 (0.52–1.29)	0.390	0.80 (0.51–1.27)	0.350	0.78 (0.49–1.24)	0.291
Quartile 3	153	70	0.57 (0.36–0.92)	0.020^*^	0.57 (0.35–0.91)	0.018^*^	0.57 (0.35–0.92)	0.022^*^
Quartile 4	137	57	0.50 (0.31–0.81)	0.005^**^	0.50 (0.31–0.81)	0.005^**^	0.52 (0.32–0.86)	0.010^*^
*p* for trend				0.002^**^		0.002^**^		0.004^**^
One SD increase			0.71 (0.56–0.89)	0.003^**^	0.70 (0.56–0.89)	0.003^**^	0.73 (0.57–0.92)	0.008^**^

Model 1: Adjusted for gender, age, and BMI. Model 2: Adjusted for gender, age, and DM. Model 3: Adjusted for gender, age, BMI, DM, TG, TC, LDL-C, and HDL-C.

CI, confidence interval. ^*^p < 0.05; ^**^p < 0.01; and ^***^p < 0.001.

## 4. Discussion

In the present study, a total of 1,068 individuals were enrolled and our results showed that OP individuals had significantly lower Hb level as compared to non-OP individuals, and the lower Hb level was related to higher OR of OP in the elderly Chinese population.

Currently, a total of 49.3 million women and 10.9 million men in China are estimated to have OP (31). Gender, age, and BMI are the major risk factors of OP. With the increase of age, the bone metabolism is impaired because of lower IGF-1 level (32), unbalanced sex hormones, lack of exercise, and so on (33). Lower BMI has been shown to have a relationship with higher risk of OP (34) which is induced by increased mechanical load on the bone with higher body weight and higher estrogen production in the adipose tissues (35). However, obesity may deteriorate bone because of ectopic adipocyte accumulation in the bone marrow (BM) cavities, and the adipocytes in BM can also release a wide variety of adipokines which may regulate bone remodeling directly or indirectly (36). Moreover, nutritional status and exercise levels can affect the correlation between Hb levels and BMD. Imbalance dietary habit can result in low protein and malnutrition, which can result in inadequate nutritional status and contribute to diseases such as anemia, hypoproteinemia, and low BMD (37). Moreover, physical exercise favors maintenance of bone mineral density during aging (38).

Anemia and OP always occur simultaneously in the elderly population. Some clinical studies have shown the relationship between anemia and risk of OP. One study conducted in Korea indicated a positive relationship between blood cell count and BMD in the healthy postmenopausal women which suggests that blood cell count may serve as a putative marker for estimating BMD (39). Another study on 371 postmenopausal women (including 82 anemic patients) showed that anemia was one of the risk factors for low BMD in the postmenopausal women (40). Moreover, a longitudinal study based on a large nationwide population showed that patients with a history of iron deficiency anemia (IDA) had a near two-fold risk for OP (41).

With the increase of age, the decrease of Hb may be caused by the decline of red blood cell production due to hematopoietic dysfunction including reduced regenerative capacity and myeloid-biased differentiation (1, 42), the shortened survival of red blood cells (43), nutritional deficiency, and impaired inflammatory processes (44). However, recent studies also reveal direct connection between bone metabolism and hematopoiesis. Firstly, hematopoiesis origin from bone marrow which facilitates structural support and provides sites for hematopoiesis (45). Secondly, osteoblastic lineage cells, as a part of hematopoietic stem cell niche, have been shown to support bone marrow hematopoietic stem and progenitor cells (HSPCs) in mice (46, 47). Other findings also indicate that bone marrow hematopoietic dysfunction may affect bone metabolism (48), Moreover, chronic hypoxia and its related oxidative stress (49, 50) due to lower Hb level may directly interfere with bone mass and bone metabolism (51).

There were several strengths in the present study. The individuals who had no diseases or did not take drugs which could influence Hb level and bone metabolism were included in this study, and thus the results may be applicable in most elderly subjects. However, there are also some limitations. (1) As a cross-sectional study, the cause-and-effect relationships cannot be fully established and long-term follow-up studies are needed; (2) We did not take into account other factors that may affect the correlation between Hb levels and BMD, such as nutritional status and exercise levels in this study; and (3) Whether the increase of Hb level after nutritional supplementation may delay the bone loss in the elderly population is needed to be determined by more randomized, controlled trials.

## 5. Conclusion

Serum Hb level is positively related to the bone mass in the elderly Chinese population and the decrease of Hb level may increase the odds ratio of OP. Thus, BMD should be monitored closely in the anemia patients.

## Data availability statement

The raw data supporting the conclusions of this article will be made available by the authors, without undue reservation.

## Ethics statement

The studies involving human participants were reviewed and approved by Ethics Committee of Shanghai Tongji Hospital. The patients/participants provided their written informed consent to participate in this study.

## Author contributions

YL designed the work, acquired and analyzed the data, and participated in writing the manuscript. YZ, JL, XZ, ZZ, HL, PL, BM, and YG acquired and analyzed the data and revised the manuscript. LS contributed to the concept and design of the work, and reviewed and revised the manuscript. All authors contributed to the article and approved the submitted version.

## Funding

This work was supported by the Shanghai Science and Technology Foundation to LS (19ZR1448600); Project supported by Shanghai Commission of Science and Technology to LS (22Y11904600) and Clinical Research Project of Tongji Hospital of Tongji University to LS [Grant No. ITJ(ZD)1904].

## Conflict of interest

The authors declare that the research was conducted in the absence of any commercial or financial relationships that could be construed as a potential conflict of interest.

## Publisher’s note

All claims expressed in this article are solely those of the authors and do not necessarily represent those of their affiliated organizations, or those of the publisher, the editors and the reviewers. Any product that may be evaluated in this article, or claim that may be made by its manufacturer, is not guaranteed or endorsed by the publisher.
